# Characterization of Micro-Threaded Stem Taper Surfaces of Cementless Hip Endoprostheses

**DOI:** 10.3390/ma17112751

**Published:** 2024-06-05

**Authors:** Drago Dolinar, Boštjan Kocjančič, Klemen Avsec, Barbara Šetina Batič, Aleksandra Kocijan, Matjaž Godec, Marko Sedlaček, Mojca Debeljak, John T. Grant, Timon Zupanc, Monika Jenko

**Affiliations:** 1 Department for Orthopaedic Surgery, University Medical Centre Ljubljana, Zaloška 9, 1000 Ljubljana, Slovenia; dolinardrago@gmail.com (D.D.); kocjancicb@gmail.com (B.K.); kavsec@gmail.com (K.A.); zupanc.timon@gmail.com (T.Z.); 2 Orthopaedic Surgery Chair, Faculty of Medicine, University of Ljubljana, Vrazov trg 2, 1000 Ljubljana, Slovenia; 3 Institute of Metals and Technology, Lepi pot 11, 1000 Ljubljana, Slovenia; barbara.setina@imt.si (B.Š.B.); aleksandra.kocijan@imt.si (A.K.); matjaz.godec@imt.si (M.G.); marko.sedlacek@imt.si (M.S.); 4 University Rehabilitation Institute Republic of Slovenia, Linhartova 51, 1000 Ljubljana, Slovenia; mojca.debeljak@ir-rs.si; 5 Research Institute, University of Dayton, Dayton, OH 45469, USA; john.grant@surfaceanalysis.org; 6 MD-RI Institute for Materials Research in Medicine, Bohoričeva 5, 1000 Ljubljana, Slovenia; 7 MD Medicina, Sanatorium, Bohoričeva 5a, 1000 Ljubljana, Slovenia

**Keywords:** total hip arthroplasty, stem micro-threaded taper, taper surface morphology, microstructure, corrosion, Ti implant alloy

## Abstract

We investigated micro-threaded stem taper surface and its impact on premature failures, aseptic loosening, and infection in cementless hip endoprostheses. Our study focused on the fretting, and crevice corrosion of micro-threaded tapers, as well as the characterization of the microstructure and surface properties of two new and three retrieved Zweymüller stem tapers. The retrieved samples were selected and examined based on the head–stem taper interface being the sole source of modularity with a metallic component, specifically between the Ti alloy taper stem and the ceramic head. To determine the surface chemistry and microstructures of both new and retrieved hip endoprostheses stem taper titanium alloy, scanning -electron microscopy (SEM) was employed for morphological and microstructural analyses. Energy dispersive spectroscopy (EDS) was utilized for characterizing chemical element distribution, and electron backscattered diffraction (EBSD) was used for phase analysis. The roughness of the micro-threated stem tapers from different manufacturers was investigated using an optical profilometer, with standard roughness parameters Ra (average surface roughness) and Rz (mean peak to valley height of the roughness profile) being measured. Electrochemical studies revealed no fretting corrosion in retrieved stem tapers with ceramic heads. Consequently, three retrieved tapers and two new ones for comparison underwent potentiodynamic measurements in Hank’s solution to determine the corrosion rate of new and retrieved stem taper surfaces. The results showed a low corrosion rate for both new and prematurely failed retrieved samples due to aseptic loosening. However, the corrosion rate was higher in infected and low-grade infected tapers. In conclusion, our study suggests that using ceramic heads reduces taper corrosion and subsequently decreases the incidence of premature failures in total hip arthroplasty.

## 1. Introduction

Total hip arthroplasty (THA) procedures have achieved significant success over the decades. Kurtz et al. have reported an increase in the number of patients in the USA requiring THA and subsequent revision surgeries, attributing this trend to the aging population [1]. The number of patients who need THA is available in national registries of countries, in hospital registries, etc. The Slovenian University Medical Centre, Department for Orthopaedic Surgery, Ljubljana, has an average of 800 THA annually [2].

**Figure 1:**
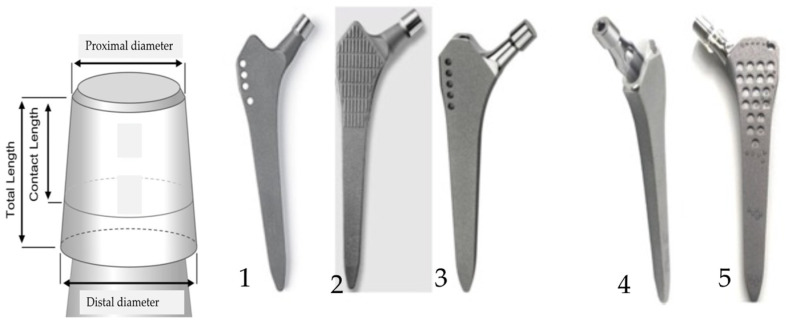
The measured taper characteristics are proximal female and distal male diameters, contact length, and total length, as shown for a stem taper at the left. The investigated cementless hip stems from different manufacturers: 1—SL-Plus, Smith & Nephew, 2—Alloclassic Varial Zimmer, 3—Alloclassic Zweymüller Zimmer, 4—Adler Ortho Modula, and 5—Lima Corporate ZM C2.

The attachment of the metal, and subsequently ceramic, head to the titanium alloy stem is accomplished using a Morse taper, which involves a male taper on the stem and a female taper within the femoral head [3,4,5]. As illustrated in Figure 1, each stem taper possesses distinct properties, including proximal diameter, distal diameter, total length, contact length, taper angle, straightness, roundness, and surface characteristics. All these values are not standardized and vary between manufacturers. Every modular connection of metal alloys in contact with body fluids and exposed to micromotion is subject to corrosion [6,7,8]. The taper’s interface is influenced by several factors, such as (i) design and material, (ii) assembly (surgical factors), and (iii) loading (patient factors) [4,7]. Urish et al. thoroughly described the fundamental principles of various types of corrosion that can affect the surface of an orthopedic implant, including pitting, crevice corrosion, fretting, and mechanically assisted crevice corrosion [6].

Cales et al. reported that each implant company adheres to its own manufacturing specifications and discussed the pros and cons of standardizing tapers in hip endoprostheses [8]. However, to date, no standardized taper regarding dimensions, metallurgy, manufacturing tolerances, or surface finish has been established by the International Organization for Standardization (ISO) or ASTM International [8]. The general standard is 12/14 (it was pioneered in the orthopedic industry by the Italian firm Cremascoli, formerly known as Adler Ortho), indicating a 12 mm diameter at the apex of the cone and 14 mm at its base. However, the exact length of the taper is not rigorously defined; thus, the angle may exhibit slight variations. It is recommended, particularly for partial dentures, that both components originate from the same manufacturer. However, this recommendation becomes less imperative during revisions, where strict adherence is not maintained. The specific shape of the taper, including notches and surface finish, remains undefined.

Currently, there are approximately 60 different types of cementless stem components for hip prostheses available on the market [8]. Understanding the behavior of each prosthesis under specific clinical conditions is crucial [8,9,10,11,12,13,14,15,16,17,18]. 

The Zweymüller cementless hip endoprosthesis, featuring the SL-PLUS^®^ femoral stem, has maintained the same design for the past 30 years. It is constructed from forged titanium Ti6Al7Nb alloy, or Ti6Al4V alloy in the USA and non-EU countries, with a double-taper straight stem of rectangular cross-section. Its grit-blasted surface, with a roughness of 2–5 µm, promotes bone ingrowth. Roškar et al. report that, to date, there has been no single-center study involving more than 2000 Zweymüller endoprostheses with over 20 years of follow-up [19,20].

The primary reasons for the premature failure of joint arthroplasty are aseptic loosening and periprosthetic joint infection [12,15]. Aseptic loosening can occur due to various factors, including implant micromotion within the bone during loading, the presence of corundum wear particles from grit-blasted surfaces, including the taper, generation of wear particles leading to inflammation and bone resorption, and subsequent formation of a suboptimal functional interface (osteointegration) between the implant and the patient’s bone [21,22,23,24,25,26].

The objective of the current study is the investigation of cementless Zweymüller stems, the femoral component, of hip endoprostheses. We were interested in the micro-threaded stem taper and its influence on premature failure, aseptic loosening, and infection. We have focused on the characterization of surface properties, micromorphology, microstructure, phase structure, and surface properties of two new (Alloclassic of Ti6Al7Nb and Lima Corporate of Ti6Al4V alloy) and three retrieved taper stems. 

We analyzed retrieved samples where the sole source of modularity involving a metallic component was the interface between the Ti6Al7Nb alloy taper stem and the ceramic head.

Peta et al. reported that surface wettability is detrimental to osteointegration and they proposed scale-dependent contact angle analyses of electro-discharge machined Ti6Al4V alloy for biomedical implant applications [27]. Wettability is very important in implant modeling. Wettability is extremely important on the stem implanted directly in the bone marrow where osteointegration between the stem made from titanium alloys (Ti6Al4V or Ti6Al7Nb) with a rough surface and bone plays the role of surgery success. The rough surface is needed for the adhesion of BMSC cells and consequently good osteointegration and long survivorship of the implant.

Stockhausen et al. documented that degradation at the modular head–neck interface in total hip arthroplasty (THA) primarily manifests as corrosion and fretting, potentially resulting in peri-prosthetic failure due to adverse reactions to metal debris [3]. Their retrieval study aimed to quantify variations in surface topographies of stem tapers and evaluate their impact on corrosion and/or fretting formation in titanium alloy stem tapers coupled with ceramic heads. They observed significant variability in surface characteristics among threaded stem tapers: Alloclassic and CLS tapers exhibited deeply threaded trapezoid-shaped profiles with thread heights exceeding 65 μm, while sawtooth-shaped Bicontact and triangular SL-Plus taper showed lower thread heights below 14 μm. Comparatively lower corrosion and fretting scores were noted in lightly threaded tapers as opposed to deeply threaded ones in combination with ceramic heads. The authors concluded that understanding the relationship between stem taper surface topography and the clinical manifestation of corrosion and fretting could enhance the performance of contemporary THAs and contribute to more durable clinical outcomes [3,4,28,29].

The aim of this study is to analyze the microstructure, surface characteristics, and microtopography of both new and retrieved 12/14 stem tapers from various manufacturers. Specifically, the focus is on retrievals where the only form of modularity involving a metallic component was the interface between the Ti alloy taper stem and the ceramic head.

## 2. Materials and Methods

### 2.1. Materials

All retrieved Ti6Al7Nb stems from the cementless Zweymüller (ZM)-type hip endoprostheses were collected as part of the register of explanted orthopedic endoprostheses at UMC Ljubljana, Slovenia, during revision surgeries. For comparison, we investigated two out of five new stems (after their expiry date) of Smith & Nephew, Alloclassic Varial, Alloclassic Zimmer, and Lima Corporate manufacturers. 

The investigation encompassed 45 stems from cementless hip endoprostheses that prematurely failed due to (i) aseptic loosening (15 implants), (ii) infection (15 implants), and (iii) low-grade infection (15 implants). The time interval between the primary hip replacement and the revision surgery ranged from 36 to 259 months for aseptic loosening, 3 to 40 months for infection, and 12 to 198 months for low-grade infection [4,13,14].

The retrieved stems were manufactured by SL-Plus, Smith & Nephew (London, UK), and Endoplus (London, UK). After revision surgery, the implants were sent for sonication and microbiological analysis in Ringer’s solution, followed by cleaning and sterilization [4,5,6,7].

**Figure 2:**
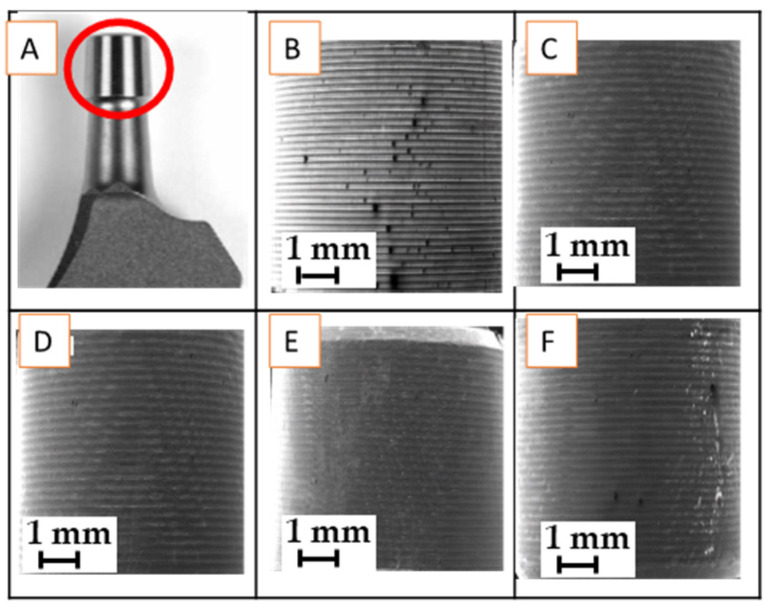
(**A**) The red circle indicates a micro-threaded 12/14 stem taper, new, Smith & Nephew, (**B**–**F**): 11× magnification SEM/SE images of different ZM 12/14 tapers and different manufacturers: (**B**) Alloclasic Varial new, (**C**) Lima new, (**D**) retrieved I-212-32 months, Smith & Nephew, (**E**) retrieved I-31-129 months, Smith & Nephew, and (**F**) retrieved A383-239 months, Endoplus.

The samples, consisting of tapers, as described in Table 1 and in Figure 2, were cut from the stems and cleaned by standard procedures. The micro-threaded taper surface of 5 selected samples, namely (i) A-283-239 month—aseptic loosening, (ii) I-212-32 month—infection, (iii) I-129-32 low-grade infection, (iv) Alloclassic Varial—new and (v) Lima Corporate—new (Ti6Al4V) were examined. The retrieved stems were cleaned according to standard procedures at University Medical Centre Ljubljana, which consisted of immersion in 2% micro soap solution, followed by acetone, isopropanol, 95% ethanol, and deionized water. Sterilization was performed by autoclaving according to a standard protocol at 120 °C and a pressure of 1.25 bar for 20 min. Afterward, sterilized stems were kept in sterile bags in a dry place for further investigation. New femoral components were cleaned and sterilized at the manufacturer’s site, and the special bags were opened on-site before the investigation.

### 2.2. Methods

The morphology, microstructure, surface chemistry, and phase composition of the Ti6Al7Nb and Ti6Al4V alloy samples were examined using a field-emission scanning electron microscope (ZEISS crossbeam 550 FIB-SEM, Carl Zeiss AG, Oberkochen, Germany). 

The instrument features secondary-electron (SE) and backscattered-electron (BE) imaging modes for morphological analysis and energy-dispersive X-ray spectroscopy (EDS) (EDAX, Octane Elite, Draper, Cambridge, MA, USA) for surface chemistry analysis to a depth of approximately 3 µm. SE and BE imaging were conducted at an acceleration voltage of 15 kV and a current of approximately 1.5 nA under a vacuum of less than 10^−6^ mbar.

For phase analysis and grain orientation, the instrument is equipped with an electron backscattered diffraction analyzer, EBSD, with Hikari Super Plus camera with ApeX (Edax) software 25-2021, and data were analyzed in OIM (Edax). 

The micro-threaded surface roughness of stem tapers was measured using an optical profilometer made by Alicona Infinite Focus G4 (Raaba-Grambach, Austria). The standard roughness parameters *R*a—average surface roughness and *R*z—mean peak to valley height of roughness profile were utilized for this investigation. Additionally, profile depth and peak spacing for different samples were examined, with an evaluation length of 2.67 mm for all samples.

The micro-threaded surface roughness of stem tapers was measured using an optical profilometer made by Alicona Infinite Focus G4 (Raaba-Grambach, Austria) using focus variation method of measurement. A 2 × 1 field of view resulting in an area of 2681 × 1088 mm was captured using a 10× zoom lens. From the obtained 3D profile, the 2D roughness parameters were calculated in the longitudinal direction in accordance with the ISO 4287 standard [30]. The standard roughness parameters *R*a—average surface roughness and *R*z—mean peak to valley height of roughness profile were utilized for this investigation. Additionally, profile depth and peak spacing for different samples were examined, with an evaluation length of 2.67 mm for all samples.

To measure the corrosion rate, an electrochemical study was conducted in simulated physiological Hank’s solution at 37 °C and pH 7.8 using a BioLogic Modular Research Grade Potentiostat/Galvanostat/FRA Model SP-300 (BioLogic Science Instruments, Seyssinet-Pariset, France) with EC-Lab Software V11.27 (EC-Lab V11.27). The experiments were performed in a three-electrode cell configuration, with the tested specimens as the working electrode (WE), a saturated calomel electrode (SCE, 0.242 V vs. SHE) as the reference electrode (RE), and a platinum net as the counter electrode (CE). The samples were stabilized at the open-circuit potential (OCP) for 1 h before the electrochemical measurements were made. The potentiodynamic curves were recorded with a scan rate of 1 mVs^−1^ from −250 mV vs. SCE relative to the OCP [31].

All the measurements were repeated three times.

## 3. Results 

### 3.1. Microstructure of Ti6Al7Nb and Ti6Al4V Stem Tapers

**Figure 3:**
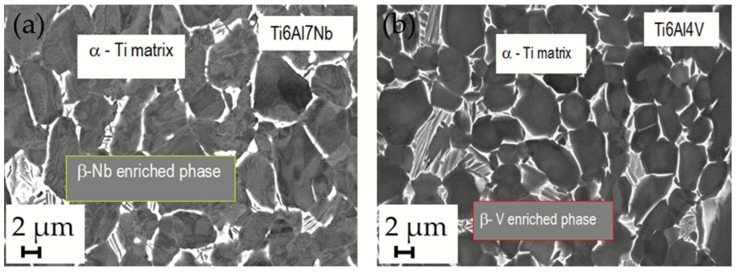
(**a**) SEM/BE image of Ti6Al7Nb alloy microstructure (A283). It consists of two phases (α + β). α-phase (dark) represents the matrix, which has a close-packed hexagonal crystal structure (hcp), and the β-Nb-enriched β phase (white) in the body-centered cubic structure. (**b**) SEM/BE image of Ti6Al4V alloy microstructure of new Lima stem. α phase (dark) represents the matrix and the β-V-enriched phase (white) at M2500.

**Figure 4:**
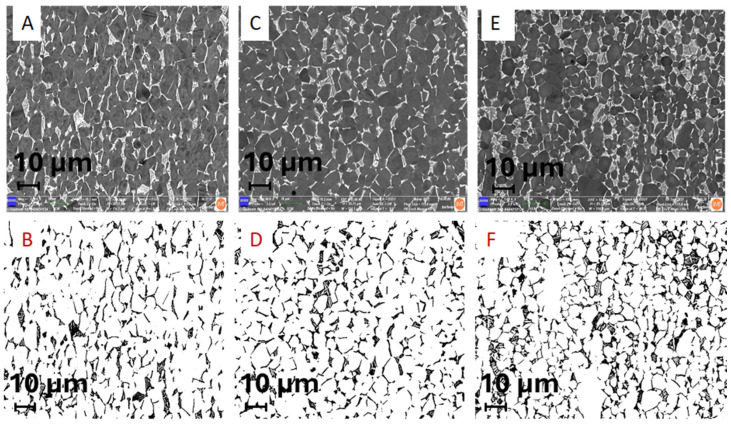
(**A**,**B**) BE/SE images of dual-phase microstructure (α + β) and their distribution of a stem taper retrieved after 239 months (ENDOPLUS, London, UK) that prematurely failed due to aseptic loosening Ti6Al7Nb alloy A-283, M1000; (**C**,**D**) SEM/BE images of dual-phase microstructure (α + β) and phase distribution in stem taper that prematurely failed after 3 months due to infection, I-31 Smith & Nephew (London, UK), M1000, (**E**,**F**) SEM/BE images of dual-phase microstructure α + β and their distribution of new stem taper Lima, made of Ti6Al4V alloy; M1000 β-phase in the row below (b, d, e) is black. The amount of α (Ti, Al, O, N, matrix) and β (Nb- or V-enriched phase) is represented in Table 2.

**Figure 5:**
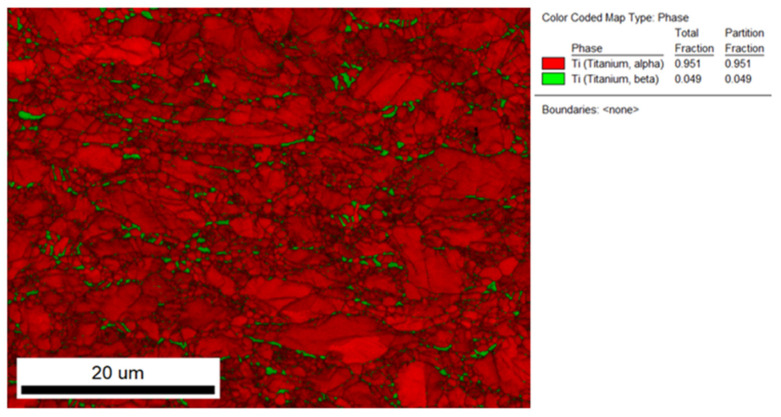
EBSD image of Ti6Al7Nb new sample. α phase is Ti matrix in hcp structure, red, and with Nb-enriched β phase, bcc structure is green.

The microstructure of the stem taper of the implants manufactured from Ti alloys (Ti6Al7Nb and Ti6Al4V) is shown in Figure 3. A dual-phase microstructure is observed in the matrix, Ti phase is α-Ti (dark grey), and the Nb-rich or V-rich phase (light grey) is β-Ti (Figure 4). The dual-phase structure is confirmed using the EBSD method (Figure 5).

### 3.2. The Estimation of Microstructure Alpha- and Beta-Phase Contents in Ti6Al7Nb and Ti6Al4V Alloys

Ti implant alloys have a dual-phase microstructure (α + β). Their distribution was estimated using a field emission scanning electron microscope by SE (secondary electron image) and BE (backscattered electron image). Results are shown in Figure 4 and Table 2.

The results of the microstructure analysis of selected retrieved and two new samples for comparison (Table 2) showed that according to the standards [32,33] (ASTM F-1295 Ti6Al7Nb and ASTM F-136 Ti6Al4V), the dominant phase, α phase (matrix), with 85 to 92% and 8 to 15% β-Nb-enriched phase of retrieved samples and 79 to 89 α phase and 11 to 21% of β phase of new samples consisted of the same alloy Ti6Al7Nb. We compared the microstructure analysis with the Ti6Al4V alloy, where we found 84% α phase, matrix, and V-enriched β phase at 16%. Of all the investigated samples, only the I-212 stem taper that prematurely failed after 3 months due to infection of the hip endoprostheses differs slightly in microstructure with the lowest Nb-enriched β phase. Besides the material properties, such as microstructure, the survivorship of cementless hip endoprostheses is very complex and depends on several factors, such as the impact on surgeons, the patient, materials, contamination, etc. 

### 3.3. SEM/EBSD Characterization of Stem Taper Phase Microstructure

The quantitative determination of phase distributions in the new sample Ti6Al7Nb alloy is possible by using the electron backscattered diffraction integrated technique on the field emission scanning electron microscope, EBSD, where the stem taper microstructure is (α + β), as in Figure 5. The EBSD method is compatible with the XRD method but on a smaller scale and for that reason is mounted on the FE-SEM. The EBSD method is very precise; the limitation of EBSD, besides being a very accurate, precise method, is that it is very time-consuming, and for that reason is suitable for basic research and the development of new biomaterial alloys.

The microstructures of all the investigated stem tapers manufactured from Ti6Al7Nb alloy are similar. We compared the stem taper’s microstructure of Ti6Al4V and found that the latter is more small-grained, which is already known from several investigations [17].

### 3.4. SEM/EDS Characterization of Stem Taper New Implants’ Microstructure

**Figure 6:**
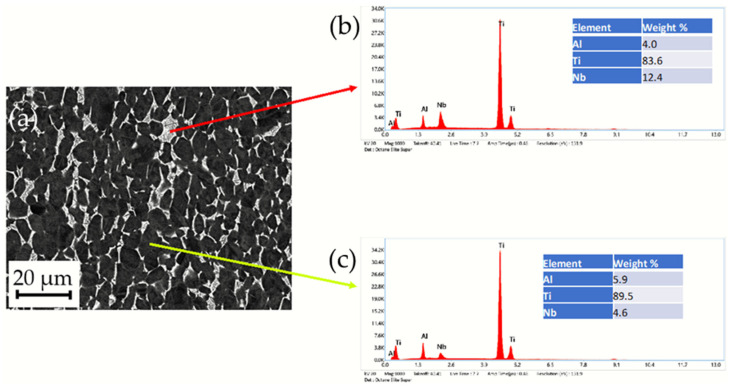
(**a**) SEM/BE images of A-283, Ti6Al7Nb alloy, stem taper of the hip endoprosthesis, survivorship 239 months, aseptic loosening, microstructure dual phase is observed; (**b**) red arrow shows Nb enriched β phase; (**c**) yellow arrow shows the matrix Ti6Al7Nb alloy.

Two new tapers were investigated for comparison with the retrieved, prematurely failed implants. The results were similar, so we represent one sample, with the longest survivorship A283 in Figure 6.

The SEM/EDS images of the surface chemistry are shown in Figure 6. The selected stem taper with a survivorship of 239 months prematurely failed due to aseptic loosening. The typical (α + β) dual-phase Ti6Al7Nb alloy is shown in Figure 6a. Figure 6b shows the distribution of Ti in the sample, (c) EDS mapping shows the distribution of Al in the microstructure, and (d) SEM/EDS mapping shows the distribution of Nb. We may conclude that the major dark-grey phase is the Ti matrix, α phase, and the light-grey phase, enriched with Nb is the β phase. 

**Figure 7:**
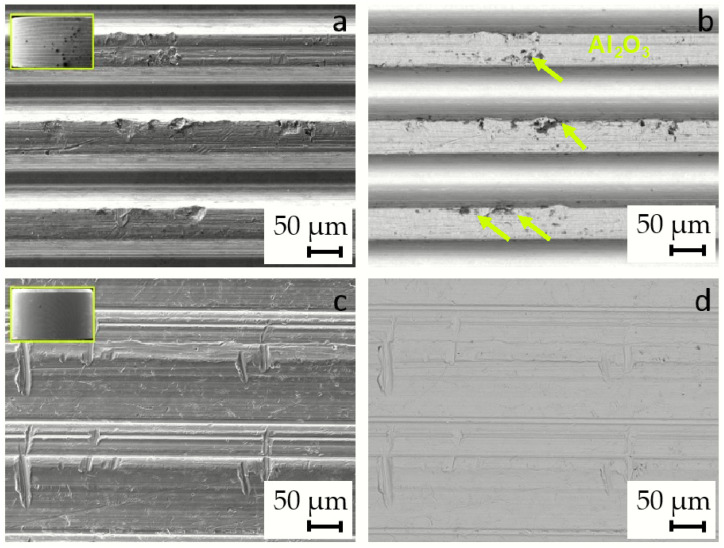
Images of the surface of the new, unused tapers. (**a**) SE image of Alloclassic new; (**b**) BE image of Alloclassic new; the remains of grit blasting are observed, dark particles, marked with yellow arrows; (**c**) SE image of Lima Corporate new; and (**d**) BE image of Lima Corporate new.

The images of the new, unused implants show some signs of abrasive damage and wear, which is attributed to the manufacturing process, as seen in Figure 7. Some dark particles are visible in the backscattered electron images (BE), in the case of the Alloclassic implant (Figure 7b), while in the case of the Lima Corporate implant taper, the surface seems rough, but no particles are visible in the (sub)surface area.

**Figure 8:**
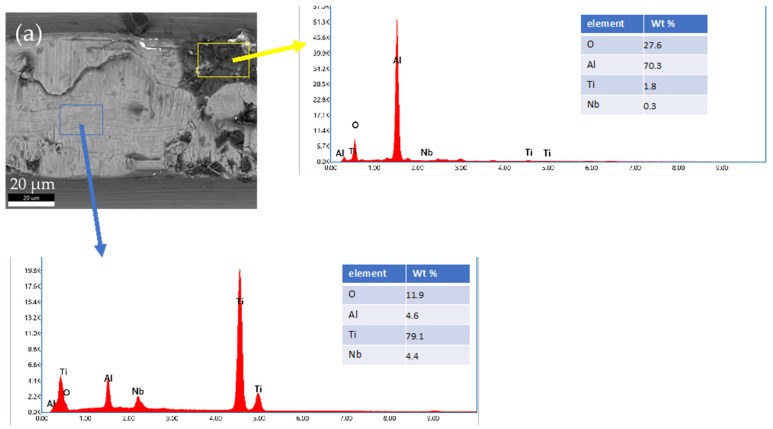
EDS analysis of a selected area of new Alloclassic taper. (**a**) BE image of the area. Yellow box indicates the remnants of Al_2_O_3_ (corundum) of micro-threaded blaster finishing, and blue box shows the matrix Ti6Al7Nb alloy.

To identify the dark particles in the BE image of the Alloclassic taper part of the implant, we performed SEM/EDS mapping of a selected area. As is evident from the image in Figure 8a, the black particles are remnants of corundum (Al_2_O_3_) sandblasting that was performed as a finishing step in the taper manufacturing process. Additionally, the dual microstructure of the titanium alloy can be seen.

**Figure 9:**
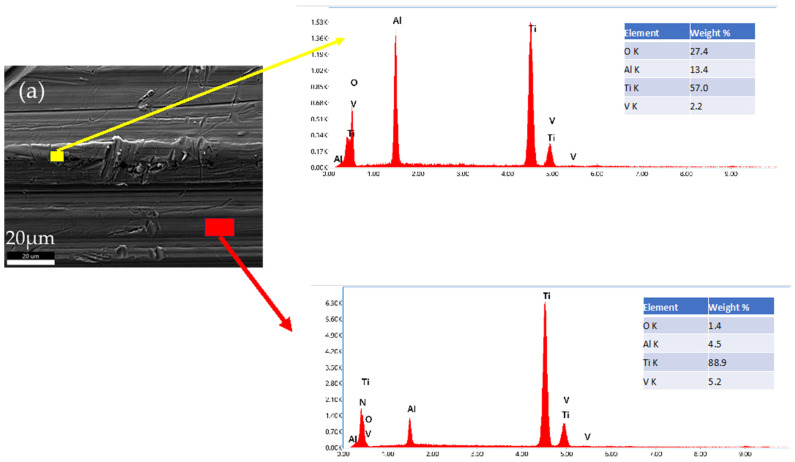
(**a**) Surface damage (abrasive wear) of a new, unused Lima Corporate taper part of an implant. EDS mapping shows evidence of corundum Al_2_O_3_ particles implanted in the material due to the manufacturing process of the implant.

On the other hand, the new implant Lima Corporate does not show larger dark particles that are visible in the backscattered electron image. The surface of the new implant nonetheless shows signs of abrasive wear, as is evident from Figure 9. 

The new, unused tapers of the implants do not have a smooth surface in both cases; there is evidence of deposited material (Al_2_O_3_) from sandblasting, and mechanical wear damage is visible in both cases. It has been shown that corundum Al_2_O_3_ sandblasting remains from the process and affects the corrosion properties and can lead to an increased number of infections [11,12]. 

### 3.5. SEM/EDS Characterization of Prematurely Failed Implants

**Figure 10:**
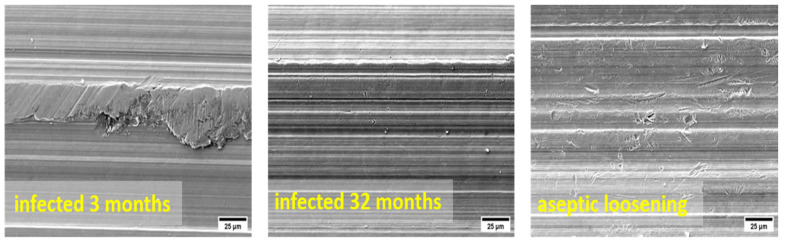
SE images of the surface of the three failed implants. The surface area shown in the figures is 230 µm × 170 µm.

Three failed implants were chosen for examination with SEM/EDS. Two implants prematurely failed due to infection, the first one after 32 months and the second after 129 months, while the third implant failed because of aseptic loosening after 239 months. The images of the surfaces of the implant taper parts are shown in Figure 10.

**Figure 11:**
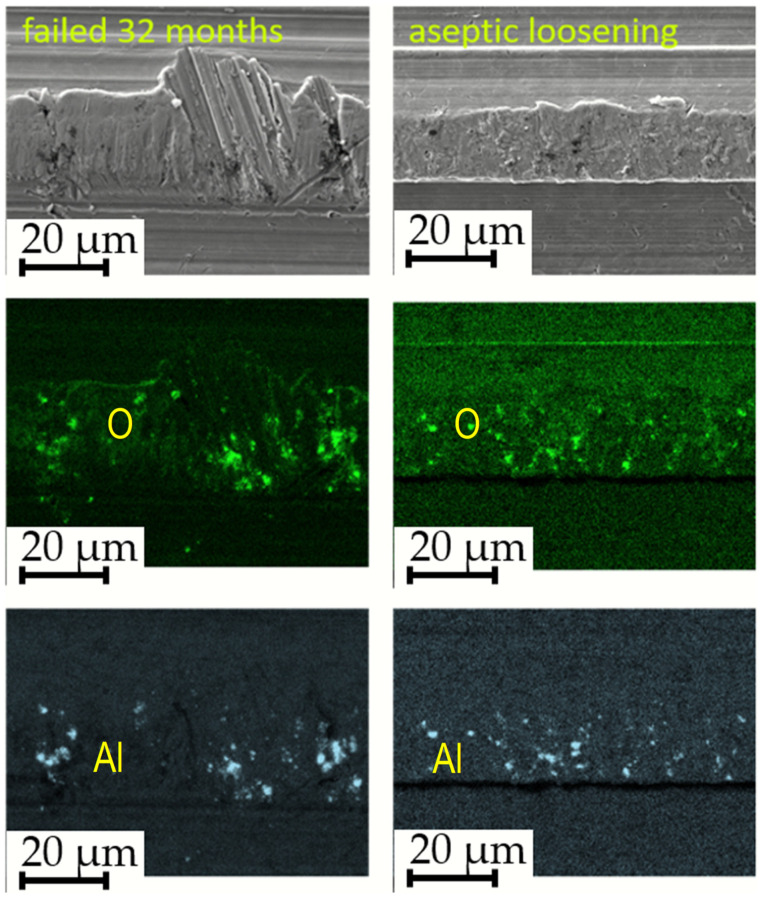
Evidence of corundum remains in an infected implant that failed after 32 months and an aseptically loosened implant after 239 months.

**Figure 12:**
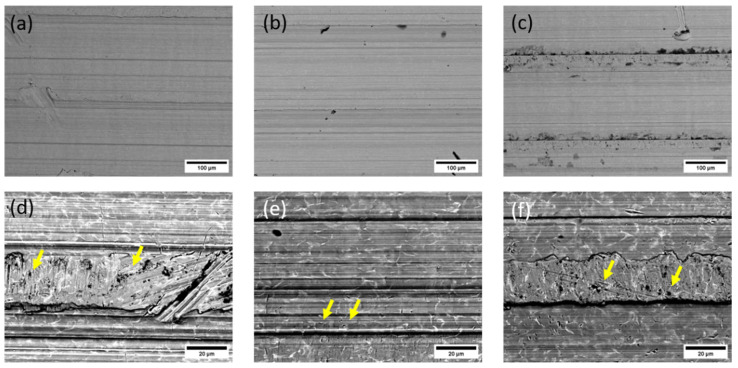
SEM/Backscattered electron images (BE) of the failed implant surfaces: (**a**,**d**) infected implant, failure time 3 months; (**b**,**e**) infected implant, failure at 32 months; and (**c**,**f**) failure due to aseptic loosening after 239 months. The arrows in the lower panel point to locations of embedded corundum particle clusters.

The surfaces of prematurely failed implants show signs of abrasive wear, like the new, unused implants, mainly due to machining and further sandblasting, as seen in Figure 11 and Figure 12. No cracks or oxidation products are visible on the implant surfaces. Additional abrasive wear (most prominent in the case of the implant that failed due to aseptic loosening) could also be attributed to the revision surgery.

EDS mapping showed that there are some remnants of corundum in the subsurface layers of the failed implants, as is seen in Figure 11. The corundum remains are clustered, and the particle sizes are up to approximately 10 µm in diameter, with a similar distribution compared to the new Lima implants. No large particles (such as in the case of a new Alloclassic implant) were found on the examined surfaces of the failed implants. This is most evident from large-scale backscattered electron images, shown in Figure 12, upper panel. Only at higher magnification (lower panel of Figure 12, it is possible to distinguish individual clusters of small, embedded corundum particles. In all implants, no signs of corrosion or cracks due to fretting were observed.

### 3.6. Stem Taper Morphology: Surface Roughness

**Figure 13:**
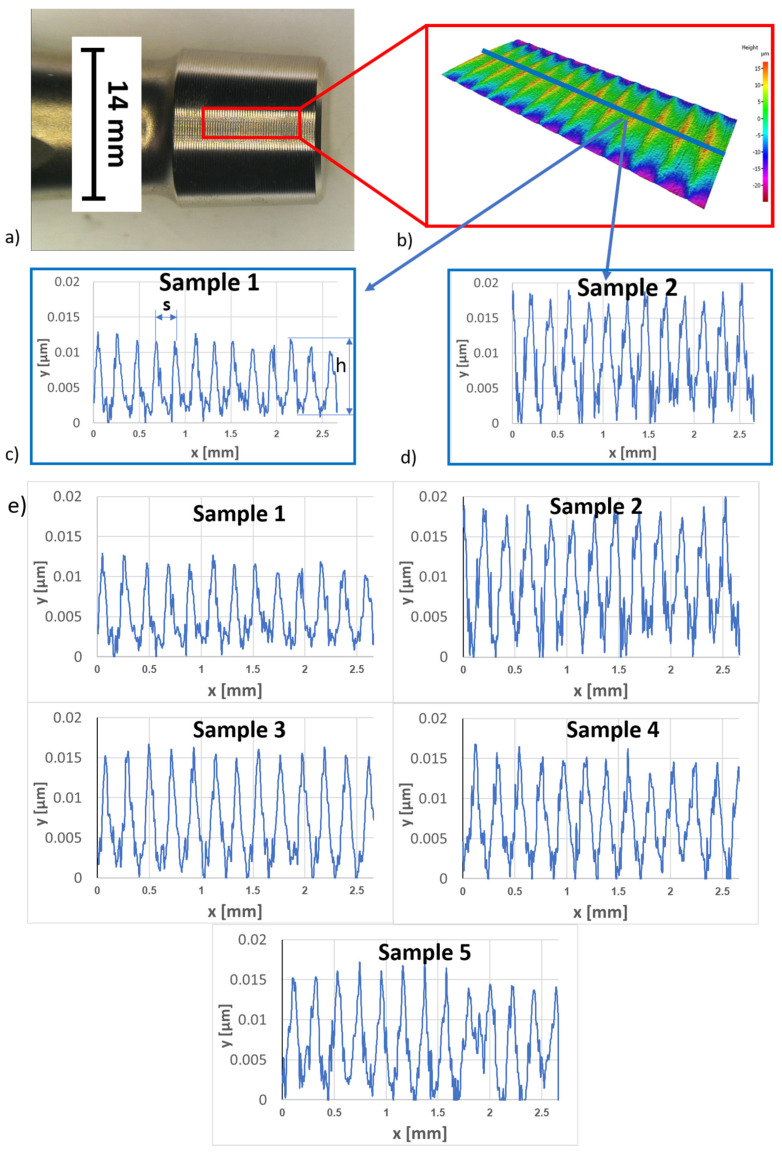
(**a**) Image of male stem taper, (**b**) 3D pseudo-profile picture, (**c**) 2D roughness profile of stem A, (**d**) 2D roughness profile of stem B, and (**e**) roughness of all five investigated stem tapers collected in Table 3.

Five samples were investigated as seen in Table 3: samples 1 to 5, two new (Alloclassic Varial-AV, and Lima Corporate) and three retrieved (A-283, I-212, and I-031) with different survivorships from 3 to 239 months. Roughness profiles of stem tapers were obtained from the surface of the taper, as seen in Figure 13. Peak spacing—s and profile depth—h (Figure 13c) were also measured for comparison. As can be seen from Table 3, there is no significant difference in roughness associated with a longer time of use. New samples AV and Lima have the lowest and highest values of Ra and Rz values. Samples AV and Lima also have different profile depths for the peaks (Figure 13c,d). Sample AV has an average depth of 9.3 and sample Lima 14.4 μm. (Table 3). This can be attributed to different manufacturers and thus different manufacturing methods. Retrieved samples, on the other hand result, have slightly lower values of surface roughness and profile depth, which can contribute to the deformation of the peaks. Results of surface roughness indicate that the three retrieved samples (A-238, I-212, and I-031) were manufactured using the same or similar method as sample Lima. 

### 3.7. Corrosion Properties of Investigated New and Retrieved Stem Taper Samples

**Figure 14:**
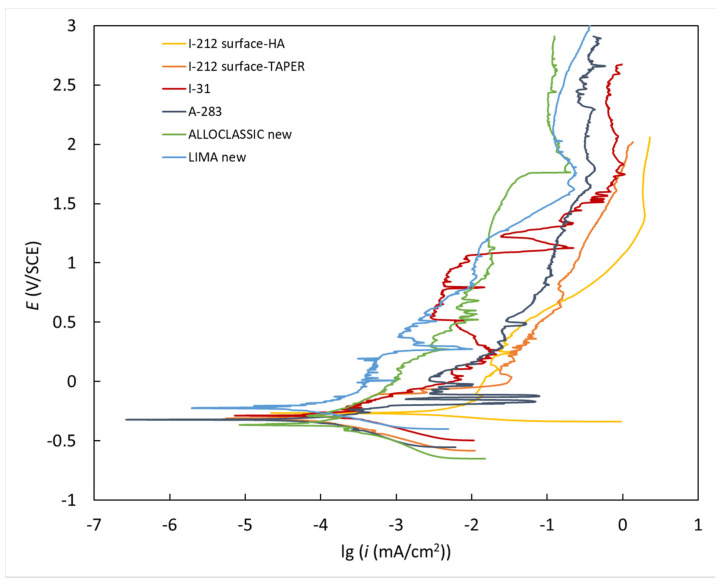
Potentiodynamic curves for the investigated new and retrieved samples were measured in simulated physiological Hank’s solution at pH = 7.8 and 37 °C.

The potentiodynamic behavior of the materials investigated in Hank’s solution is presented in Figure 14. All the materials demonstrated a broad passivation range following the Tafel region attributed to the formation of a compact outer passive film that impedes the diffusion of aggressive species, thereby enhancing the material’s corrosion resistance [31,32,33,34]. The corrosion potentials (*E*_corr_), corrosion current densities (*i*_corr_), and corrosion rates (*v*_corr_) are detailed in Table 4. The results show that the corrosion rates for both the new samples and the one with the longest exposure to real body conditions were lower compared to the other three with shorter exposure times. The implants with shorter exposure times to real body conditions were all subjected to infection, which is probably related to the formation of conditions that are detrimental to corrosion. In contrast, new implants with an intact surface as well as the implant with the longest survival in real body conditions showed an increased corrosion resistance.

## 4. Discussion

This study examined the in vivo damage on Ti-alloy micro-threaded 12/14 tapers. The damage observed in secondary implants with ceramic heads was compared to that in new implants. Both qualitative and quantitative assessments were conducted on 45 retrieved samples.

Every modular connection of metal alloys in contact with body fluids and exposed to micromotion is subject to corrosion. The taper’s interface is influenced by several factors, such as (i) design and material, (ii) assembly (surgical factors), and (iii) loading (patient factors). 

Mueller et al. showed that stem taper characteristics vary significantly among various manufacturers [5].

The assembly condition significantly influences the strength of the taper connection. Minimal invasive surgery with small incisions may indirectly affect the frequency of the taper’s corrosion due to the challenges in cleaning the taper and applying suitable assembly forces in its direction.

We investigated retrievals provided by the Department for Orthopaedic Surgery of UMC Ljubljana, Slovenia, where the sole source of modularity involving a metallic component was the interface between the ceramic head and stem taper. Each stem taper has characteristic properties, such as proximal diameter, distal diameter, total length, contact length, taper angle, straightness, roundness, and surface properties, and these values are not standardized and vary between manufacturers.

We investigated the corrosion resistance using electrochemical studies, and there was no corrosion of the retrieved tapers that experienced premature failure as a result of aseptic loosening after 239 months, infection after 3 months, and low-grade infection after 32 months. Further, three retrieved tapers and, for comparison, two new tapers were exposed to potentiodynamic measurements in Hank’s solution. The results show very low corrosion rates for both new samples (Zimmer, Lima Corporate) and the retrieved sample that prematurely failed due to aseptic loosening after 239 months. The corrosion rates of the infected and low-grade infected tapers were higher. We observed small differences in the microstructure within the limits of the standards [17]. The microstructure is very important for minimizing corrosion and bio-tribo-corrosion, corrosion of the taper, etc., which may result in aseptic loosening or joint infection, the primary reasons for failure in joint arthroplasty. 

Ceramic femoral heads were not investigated in this study, and thus no conclusions can be drawn regarding potential material transfer from the taper to the ceramic head. However, Stockhausen reported a correlation between fretting and corrosion scores in both the head and the neck, suggesting that neck scores serve as a reliable indicator of degradation in the head [3,4].

In our investigation, we did not observe taper corrosion, fretting, and crevice corrosion due to the use of ceramic heads with a dominant head size of 32 mm, which agrees with the published results [3,4,35] and the fact that the Slovenian hospital protocol requires carefully cleaned, rinsed, and dried taper stems before assembly. 

Moreover, corrosion of metal alloys is a common occurrence in the human body owing to its aggressive environment, although it may not pose a clinical concern if the extent of corrosion remains minimal [5,33,36,37,38,39,40,41,42].

## 5. Conclusions

This study illustrated that the 12/14 investigated tapers exhibit not-uniformity, with variations observed in the geometry and topography of both stem and head tapers across different manufacturers and even within the same manufacturer. 

In our study of different stems from prematurely failed cementless hip endoprostheses in terms of geometry, significant discrepancies were noted in taper length, while variations in taper angle and opening taper diameter were relatively minor. 

In our study, we did not observe taper corrosion, fretting, and crevice corrosion due to the use of ceramic heads with a dominant head size of 32 mm, which agrees with the published results and the fact that the hospital protocol requires carefully cleaned, rinsed, and dried taper stems before assembly. 

Moreover, within the human body, metal alloys inevitably undergo corrosion owing to the aggressive physiological environment. However, the clinical significance of this corrosion is not inevitable, contingent upon the extent of its occurrence.

Ultimately, the findings of this study offer pertinent taper design characteristics for new components, serving as valuable reference points for retrieval studies delineating the initial conditions for experimental investigations into taper corrosion.

The next step of this work will be a detailed investigation of the chemical composition ratio of the alpha and beta phases and their effect on the corrosion resistance of Ti6Al7Nb alloys for surgical implants. 

It is obvious that further investigation of the influence of stem tapers of ZM cementless hip endoprostheses on the biological response is needed. As the dimensions of the stem taper as well as materials are not standardized and vary between manufacturers, careful and precise assembly of the femoral component and ceramic acetabular heads is required and is very important.

## Figures and Tables

**Table 1:** Investigated samples of new and retrieved stem tapers.

Number	Sample	Lifetime Month	Cause of Premature Failure	Material	Tapers
1	Alloclassic Varial	new		Ti6Al7Nb—ZM stem	12/14
2	Lima Corporate	new		Ti6Al4V—ZM stem	12/14
3	I-212	032	Infection	Ti6Al7Nb/ceramics BD-diameter 36 **	12/14
4	I-031	129	Low-grade infection	Ti6Al7Nb/ceramics BD-diameter 32 **	12/14
5	A-283	239	Aseptic loosening	Ti6Al7Nb/ceramics BF-diameter 32 *	12/14

* Ceramic head Biolox Forte, BF; ** Ceramic head Biolox Delta, BD, ZM-Zweymüller.

**Table 2:** Microstructure analysis: the estimation of investigated stem tapers’ proportion of α and β phase.

SAMPLE No/ STEM TAPER ALLOY	Survivorship of THA/Months	Magnification	Analyzed Area (μm^2^)	Content α Phase (%)	Content β Phase (%)
**A 283 02/Ti6Al7Nb**	239	250	143.05	85.32	14.68
A 283 04/Ti6Al7Nb	239	500	38.59	87.06	12.94
A 283 06/Ti6Al7Nb	239	1000	9.69	87.51	12.49
**I 212 02/Ti6Al7Nb**	3	250	143.05	92.21	7.79
I 212 04/Ti6Al7Nb	3	500	38.59	92.49	7.51
I 212 06/Ti6Al7Nb	3	1000	9.69	92.64	7.36
**I31 02/Ti6Al7Nb**	129	250	143.05	86.73	13.27
I31 04/Ti6Al7Nb	129	500	38.59	87.60	12.40
I31 06/Ti6Al7Nb	129	1000	9.69	87.62	12.38
**Alloclassic 02/Ti6Al7Nb**	new	250	143.05	80.41	19.59
Alloclassic 04/Ti6Al7Nb	new	500	38.59	78.19	21.81
Alloclassic 06/Ti6Al7Nb	new	1000	9.69	89.47	10.53
**Lima 02/Ti6Al4V**	new	250	38.59	81.86	18.14
Lima 04/Ti6Al4V	new	500	9.69	83.93	16.07
Lima 06/Ti4Al4V	new	1000	1.55	82.62	17.38

**Table 3:** Roughness parameters Ra and Rz and profile depth and peak spacing for investigated new and retrieved samples.

Stem	Sample	Ra [μm]	Rz [μm]	Profile Depth h [μm]	Peak Spac. s [μm]
A	Allocassic Varial-new	1.98	9.64	206.3	9.3
B	Lima Corporate-new	2.92	14.90	209.7	14.4
C	I-212-32 months	2.60	12.36	205.1	13.8
D	I-031-192 months	2.27	13.22	199.9	14.1
E	A-283-239 months	2.52	12.52	206.8	15.3

**Table 4:** Electrochemical parameters derived from the potentiodynamic curves.

Material	*E*_corr_ (mV/SCE)	*i*_corr_ (μA cm^−2^)	*v*_corr_ (μm Year^−1^)
I-212 surface taper 32 months	−326.6	0.14	1.21
I-031 192 months	−250.7	0.14	1.20
A-283 239 months	−284.6	0.08	0.70
Alloclassic new	−286.3	0.09	0.81
Lima Corporate new	−221.5	0.05	0.40

## Data Availability

Data are contained within the article.
